# The 96th Amino Acid of the Coat Protein of Cucumber Green Mottle Mosaic Virus Affects Virus Infectivity

**DOI:** 10.3390/v10010006

**Published:** 2017-12-25

**Authors:** Zhenwei Zhang, Liming Liu, Huijie Wu, Lifeng Liu, Baoshan Kang, Bin Peng, Qinsheng Gu

**Affiliations:** Henan Key Laboratory of Fruit and Cucurbit Biology, Zhengzhou Fruit Research Institute, Chinese Academy of Agricultural Sciences, Zhengzhou 450009, China; zhangzhenweiv@163.com (Z.Z.); liulimingv@163.com (Lim.L.); wuhuijie@caas.cn (H.W.); liulifeng@caas.cn (Lif.L.); kangbaoshan@caas.cn (B.K.); pengbin@caas.cn (B.P.)

**Keywords:** cucumber green mottle mosaic virus, assembly initiation site, infectivity, systemic symptom, coat protein

## Abstract

Cucumber green mottle mosaic virus (CGMMV) is one of the most devastating viruses infecting members of the family Cucurbitaceae. The assembly initiation site of CGMMV is located in the coding region of the coat protein, which is not only involved in virion assembly but is also a key factor determining the long-distance movement of the virus. To understand the effect of assembly initiation site and the adjacent region on CGMMV infectivity, we created a GTT deletion mutation in the GAGGTTG assembly initiation site of the infectious clone of CGMMV, which we termed V97 (deletion mutation at residue 97 of coat protein), followed by the construction of the V94A and T104A mutants. We observed that these three mutations caused mosaic after *Agrobacterium*-mediated transformation in *Nicotiana benthamiana*, albeit with a significant delay compared to the wild type clone. The mutants also had a common spontaneous E96K mutation in the coat protein. These results indicated that the initial assembly site and the sequence of the adjacent region affected the infectivity of the virus and that E96 might play an essential role in this process. We constructed two single point mutants—E96A and E96K—and three double mutants—V94A-E96K, V97-E96K and T104A-E96K—to further understand the role of E96 in CGMMV pathogenesis. After inoculation in *N. benthamiana*, E96A showed delayed systemic symptoms, but the E96K and three double mutants exhibited typical symptoms of mosaic at seven days post-infection. Then, sap from CGMMV-infected *N. benthamiana* leaves was mechanically inoculated on watermelon plants. We confirmed that E96 affected CGMMV infection using double antibody sandwich-enzyme-linked immunosorbent assay (DAS-ELISA), reverse transcription-polymerase chain reaction (RT-PCR), and sequencing, which further confirmed the successful infection of the related mutants, and that E96K can compensate the effect of the V94, V97, and T104 mutations on virus infectivity. In addition, Northern blotting showed that the accumulation of viral RNA corroborated the severity of the symptoms.

## 1. Introduction

Cucumber green mottle mosaic virus (CGMMV), a member of the genus *Tobamovirus*, is a devastating virus infecting plants of the family Cucurbitaceae. It was first reported as cucumber virus 3 by Ainsworth in 1935 [1]. Since then, it has been identified in 43 countries and regions, and it has disseminated more quickly over the past 10 years [2]. CGMMV is naturally found in cucumber, squash, melon, watermelon, snake gourd, bottle gourd, and muskmelon [3]. In addition to cucurbits, CGMMV can also induce local lesions in *Chenopodium amaranticolor* and/or *Datura stramonium* and cause mottling and mosaic in tobacco [4]. The typical symptoms of CGMMV are leaf mottling, blistering, and distortion with stunted growth. It can induce serious internal discoloration (dirty red coloration) due to water-soaking and loss in the dietary value of watermelon [5]. Similar to other tobamoviruses, CGMMV is readily transmitted by foliage contact, handling of plants during cultivation, soil contamination, or via cucurbit seeds and pollen, and irrigation [6,7]. No insect vector is known to specifically transmit the virus. In addition, CGMMV causes serious diseases and considerable economic losses worldwide. The highest recorded average incidence of CGMMV was 58.1% in a field survey of cucurbit crops in Khyber Pakhtunkhwa Province, Pakistan [8]. CGMMV occurred in 463 hectare (ha) of watermelon cultivation regions in Korea in 1998 [9]. Studies on the effect of this virus on yield indicated that approximately 15% loss occurred due to early infection [10]. Thus, CGMMV is emerging as a major threat to plants of the family Cucurbitaceae.

Biological measures, seed treatment [11,12], molecular breeding, cultural management [13], and chemical methods [14] have been used to control the virus as components of CGMMV management. Cross-protection is an attractive biological approach for protecting crops against CGMMV. An attenuated strain (SH33b) of CGMMV has been successfully applied to muskmelon in Japanese greenhouses [15]. Molecular analysis of SH33b showed that only five of nine base changes caused amino acid substitutions [16]. Similarly, another attenuated strain VIROG-43M has been characterized which can be used for cucumber protection, and only three missense mutations that could be crucial for attenuation compared VIROG-43M with pathogenic strains MC-1 and MC-2 [17]. Thus, attenuated strains of CGMMV can be constructed by modifying the viral genome. Our previous study about point mutations at residue 284 of CGMMV replicase for exploring the way for the design of new stably-attenuated CGMMV isolates [18]. Indeed, studies on the relationship between infectivity and these mutations have received a major impetus from the perspective of CGMMV pathogenesis.

The single-stranded, positive-sense RNA genome of CGMMV contains four open reading frames encoding 186, 129, 29, and 17.3 K proteins. The 186 K protein is the read-through product of the 129 K protein, both of which are viral replicases. The other two proteins correspond to the movement protein (MP) and coat protein (CP) [19]. It is well known that virion CPs are multifunctional, with roles in replication, virion formation, cell-to-cell movement, vector transmission, genome activation, and symptom modulation [20]. The CP plays an important role in the long-distance movement of tobamoviruses. Furthermore, *Tobamovirus* CP identifies the assembly initiation site located in the genomic RNA in the initial steps of virion assembly, followed by virus rod elongation in the 3′ and 5′ direction [21]. The assembly initiation site of CGMMV is located in the coding region of CP gene [22]. Therefore, we assumed that the assembly initiation site and adjacent region might be correlated with virus infectivity.

Currently, reports regarding the relationship of assembly initiation site with virus infectivity are limited. In this study, we mutated the assembly initiation site on the infectious cDNA clone of CGMMV to analyze this association. Results showed that the assembly initiation site and its adjacent region and the 96th amino acid of CP affected CGMMV infectivity.

## 2. Materials and Methods

### 2.1. Plants and Reagents

Tobacco (*Nicotiana benthamiana*) was used as the host plant in *Agrobacterium*-mediated infiltration of this study. Healthy or inoculated *N. benthamiana* plants were incubated in a growth chamber at 28 °C and under a 16 h light/10 h dark cycle. The watermelon (*Citrullus lanatus*) strain Zhengkang No. 2 was subjected to sap-mechanical inoculation. *Escherichia coli* strain TOP10 and *Agrobacterium tumefaciens* GV3101 stocks from our laboratory were used. Commercially available antibodies of CGMMV were obtained from ADGEN (Auchincruive, Scotland, UK) for double antibody sandwich enzyme-linked immunosorbent assay (DAS-ELISA). Roche’s (Basel, Switzerland) DIG northern starter kit and Hybond-N membranes from Amersham Biosciences (Little Chalfont, UK) were used for Northern blot.

### 2.2. Construction of Mutants

All mutants in this study were constructed by site-directed mutagenesis and direct polymerase chain reaction (PCR) according to the instructions of the Fast Mutagenesis System (Transgen Biotech Inc., Beijing, China). The detailed procedure of the construction of the CGMMV clones has been described in previous study [18]. The wild type infectious cDNA clone of CGMMV was used as a template for PCR amplification to generate E96A, E96K, V97 (deletion mutation at residue 97 of coat protein), V94A and T104A mutations. Then, V94A, V97, and T104A were used as starting templates to construct the V94A-E96K, V97-E96K, and T104A-E96K mutants, respectively. V94A-E96K, V97-E96K, and T104A-E96K have a second mutation at residue 96 of the gene encoding CP. The primers containing Glu to Lys substitutions at residue 96 of the CP gene are shown in Table 1.

### 2.3. Inoculation

The mutated plasmids were transferred into *A. tumefaciens* strain GV3101 by the freeze–thaw method. Agroinoculation of *N. benthamiana* plants (6–8 leaf stage) was performed by infiltration of the top two or three fully-expanded leaves of 30-day old plants. Bacteria bearing the wild type (C284R) gene were inoculated as a positive control at the same time. For every inoculum, 12 individual *N. benthamiana* plants were inoculated and assayed thrice. 

Watermelon leaves were mechanically inoculated with the sap of CGMMV-infected *N. benthamiana* leaves. *N. benthamiana* leaves infected with the mutant virus were ground in 0.01 M phosphate buffer (pH 7.4). Fine silicon dioxide powder was dusted on both cotyledons before mechanical inoculation and at the 1–2 fully expanded leaf stage. For every inoculum, six individual watermelon plants were mechanically inoculated. After inoculation, the plants were grown in a growth chamber at 26 °C with a 14 h light/10 h dark photoperiod.

### 2.4. Detection of Mutant Infectivity

CGMMV accumulation was determined using DAS-ELISA at 7, 14, 21 days post-inoculation (dpi) according to the method of Liming et al. [18]. Similarly, DAS-ELISA was carried out with systemic leaves 10, 17, and 24 days after mechanical sap inoculation. Northern blot was performed to detect the accumulation of CGMMV RNA. Total RNA was exacted from *N. benthamiana* and watermelon using the Trizol (TAKARA RNAiso Plus, Tokyo, Japan) reagent according to the manufacturer’s instructions. A pair of primers (6212F: GGTAGTCTGGTCAGAGGCTAC; T7-3UTR: TAATACGACTCACTATAGGGTGGGCCCCTACCCGGGGAA) were designed to synthesize the probe for detecting the CGMMV RNA. The probe was complementary to 6212–6423 nucleotides of the CGMMV genomic RNA. Then, the hybridization signal was imaged using the chemiluminescence imaging system (Tanon, Shanghai, China). The instructions of the DIG Northern Starter kit (Roche) were followed for northern blotting. MP-SP (ATGTCTCTAAGTAAGGTGTCAG) and 3’UTR-AP (TGGGCCCCTACCCGGGGAA) were used as primers for RT-PCR (~1.4 Kb) according to manufacturer’s recommended procedure (TaKaRa, Tokyo, Japan). The mutated sequences were confirmed by sequencing.

## 3. Results

### 3.1. The Mutants V94A, V97, and T104A Show a Delay in Appearance of Symptoms

**Figure 1:**
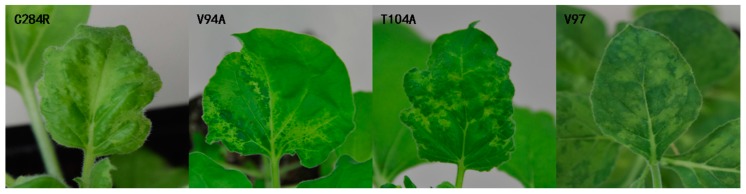
Symptoms of *N. benthamiana* inoculated with WT, V94A, T104A and V97 mutants at 10, 14, 21, and 40 days post-inoculation (dpi).

The single-site mutants were agroinoculated in the host plant *N. benthamiana*. Plants inoculated with the WT infectious cDNA clone were the first to show mosaic symptoms at 7 dpi, which were prominent at 10 dpi. In contrast, no symptoms were observed on the upper uninoculated leaves of *N. benthamiana* inoculated with mutants V94A, V97, and T104A till 14 dpi or later (Figure 1). All the upper uninoculated leaves of the mutants that exhibited symptoms and the inoculated leaves were positive by DAS-ELISA (Table 2). The different appearance times of systemic symptoms and the results of DAS-ELISA suggested that the single amino acid mutants V94A and T104A and the deletion mutant V97 were able to infect *N. benthamiana*; however, they showed an obvious delay in the appearance of symptoms. These results also showed that the assembly initiation sites and the adjacent region affected the infectivity of CGMMV.

We next analyzed the sequence of the mutants by PCR and sequence analysis of the progeny viral RNA extracted from the upper leaves of infected *N. benthamiana*. Sequence analysis of three isolates revealed that the mutation was maintained in CPs isolated from the upper uninoculated leaves of *N. benthamiana* inoculated with mutants V94A, V97, and T104A. However, we found a spontaneous and non-anticipatory glutamate to lysine mutation in the 96th residue of CP the second time. Since the E96K mutation was not strictly necessary for the initial steps in the pathogenesis of the V94A, V97, and T104A mutants. However, the E96K mutation may play a role in the later steps of pathogenesis of the V94A, V97, and T104A mutants since the three mutants exhibited a common phenotype.

### 3.2. The 96th Amino Acid of CP Affects CGMMV Infectivity

**Figure 2:**
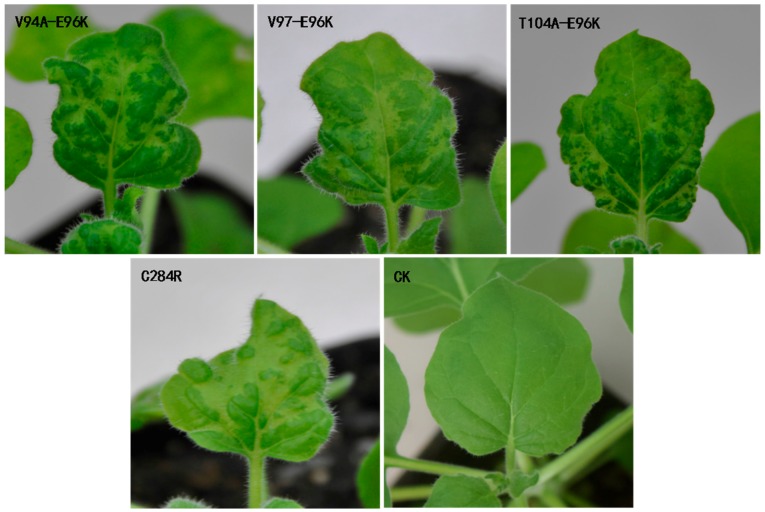
Symptoms of three double mutants V94A-E96K, V97-E96K, and T104A-E96K after inoculation at 10 dpi.

To investigate the possible roles of E96K in the pathogenesis of the V94A, V97, and T104A mutants, we constructed three double mutants—namely, V94A-E96K, V97-E96K and T104A-E96K—which were agroinoculated into *N. benthamiana* for testing their infectivity. We observed that the three double mutants showed mosaic symptoms on the upper uninoculated leaves of *N. benthamiana* at 7 dpi and the symptoms were obvious at 10 dpi (Figure 2). We next confirmed the presence of CGMMV by DAS-ELISA. In addition, we amplified the gene encoding CP from the total RNA extracted from the upper leaves of infected plants by RT-PCR. Sequence analyses showed that there were no other mutations. These results indicated that E96K facilitates the infectivity of V94A, V97, and T104A mutants.

**Figure 3:**
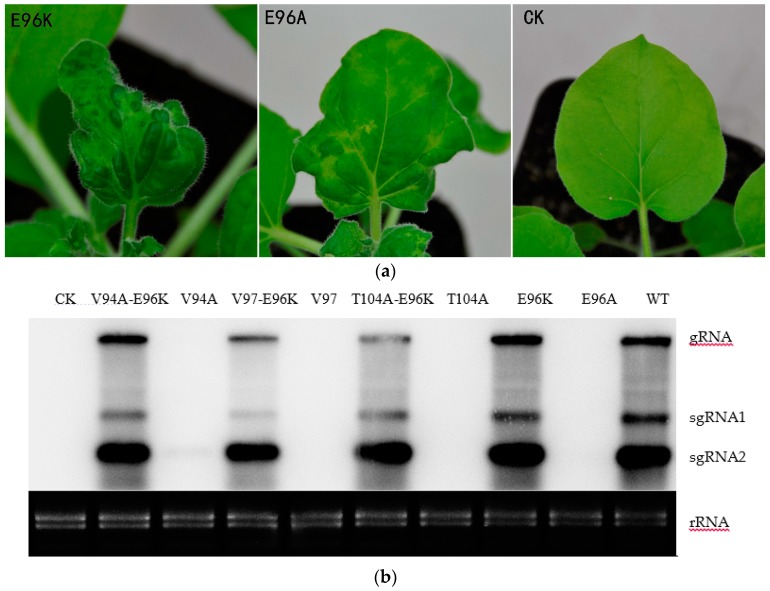
Northern blot analysis and the symptoms of single-site mutants in the E96 residue. (**a**) Symptoms of E96K and E96A at 10 and 14 dpi in tobacco, respectively; (**b**) Northern blot analysis of V94A-E96K, V94A, V97-E96K, V97, T104A-E96K, T104A, E96K, E96A, and the WT at 7 dpi. The positions of viral genomic RNA and the two subgenomic RNAs are displayed as gRNA, sgRNA1, and sgRNA2.

**Figure 4:**
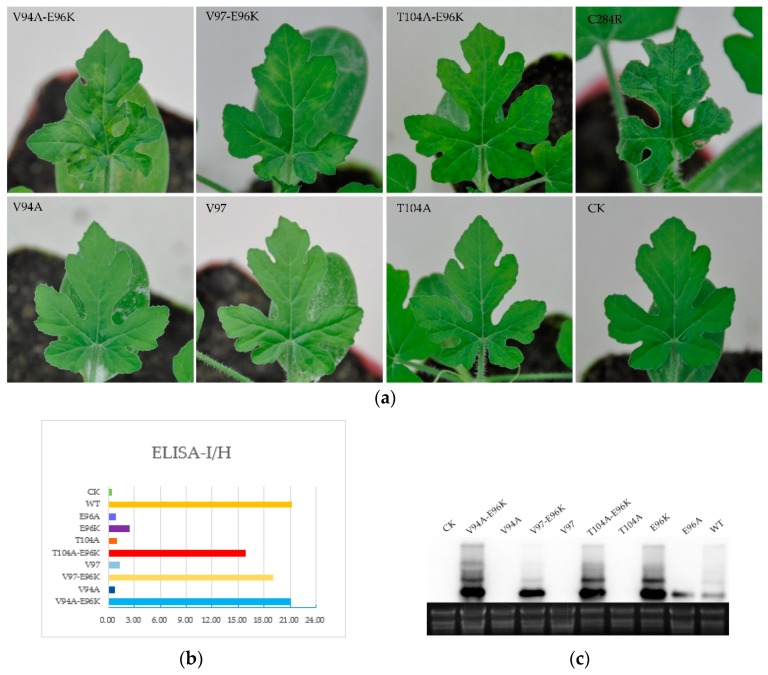
The relationship between the accumulation of mutant RNA and symptoms in watermelon. (**a**) Symptoms of watermelon inoculated with V94A, V97, T104A, V94A-E96K, V97-E96K, T104A-E96K, and C284R (WT) infected *N. benthamiana* leaves at 13 dpi; (**b**) CGMMV accumulation was determined using DAS-ELISA at 10 dpi. The abscissa displays the relative values of I/H: (sample-blank)/(negative control-blank). For all of the samples, an adjusted value of 3.0 or above was considered positive; (**c**) Northern blot analysis of V94A-E96K, V94A, V97-E96K, V97, T104A-E96K, T104A, E96K, E96A, and the WT at 17 dpi.

To further understand the role of the E96 residue on pathogenesis, we constructed the E96K and E96A mutants, which have a single amino acid change at residue 96 of the CGMMV CP. The E96A mutant exhibited mosaic in systemic leaves at 9 dpi, which became conspicuous at 14 dpi, whereas the E96K mutant showed systemic symptoms at 7 dpi and obvious mosaic at 10 dpi, which is similar to that observed with the WT. This indicated that E96 affected the infectivity of CGMMV; however, the infectivity of the E96K mutant did not show obvious changes (Figure 3a). Next, we tested whether E96 affected the infectivity of CGMMV in watermelon, as observed in *N. benthamiana*. The sap of CGMMV-infected *N. benthamiana* leaves—which included the mutants V94A, V97, T104A, V94A-E96K, V97-E96K, T104A-E96K E96K, and E96A—was mechanically inoculated into watermelon. Consistent with previous reports, the mutants V94A-E96K, V97-E96K, and T104A-E96K and the WT first showed mosaic symptoms in watermelon at 8–10 dpi, while V94A, V97, and T104A showed mild mosaic symptoms at 20 dpi or later. Figure 4a shows the systemic symptoms of the upper new leaves at 13 dpi. In watermelon, the infectivity of the single-site mutants changed, and systemic symptoms were significantly delayed compared with V94A-E96K, V97-E96K, T104A-E96K, and the WT. E96K and E96A also showed delayed systemic symptoms at 15 and 19 dpi when E96K exhibited typical symptoms of mosaic at 7 dpi in *N. benthamiana*. Thus, the E96K mutation appears to have a compensatory effect on the attenuating mutations V94A, V97, and T104A. In summary, the 96th amino acid of CP affected the infectivity of CGMMV.

### 3.3. The Relationship between the Accumulation of Mutant RNA and Symptoms in Host Plants

Northern blotting was performed with the CGMMV RNA to understand the relationship between the accumulation of viral RNA and symptoms of the host plants. Total RNA was extracted from the upper uninoculated leaves of *N. benthamiana* using the Trizol reagent at 7 dpi. As shown in Figure 3b, the viral genomic RNA and two subgenomic RNAs could be detected in the upper uninoculated leaves of plants inoculated with Agrobacterium carrying the WT but not the CK plasmid. Accumulation of V94A-E96K, V97-E96K, T104A-E96K, and E96K viral RNAs was detected at 7 dpi. In contrast, no visible bands were detected by northern blotting for mutants V94A, V97, T104A, or E96A. Next, we also detected the accumulation of mutant RNA in watermelon by northern blot analysis. Total RNA was extracted from the upper new leaves at 17 dpi. The viral genomic RNA and two subgenomic RNAs of V94A-E96K, V97-E96K, T104A-E96K, E96K, and the WT could be detected in the upper uninoculated leaves of plants at 17 dpi (Figure 4c). A closer look at the results indicates that viral RNA was consistent with the appearance of systemic symptoms in *N. benthamiana* and watermelon.

## 4. Discussion

On the basis of their ability to form ordered structures that encapsulate the viral nucleic acid, plant virus CPs possess multiple functions such as replication, virion formation, cell-to-cell movement, vector transmission, genome activation, and symptom modulation [20]. The CP of the genus *Tobamovirus* plays an important role in the pathogenesis of viral diseases. In a previous study, YSI/1, a mutant strain of tobacco mosaic virus (TMV), had an Asp-Val change at amino acid 19 which induced a yellow mosaic phenotype in *N. tabacum* instead of the light or dark green color [23]. A point mutation at CP residue 148 was responsible for the ability of TMV to elicit hypersensitivity reaction (HR) in *N. sylvestris* [24]. Substitutions at positions 43 and 50 enabled pepper mild mottle virus (PMMoV)-J to overcome L3 resistance [25]. Similarly, substitutions and deletion mutations were introduced in CGMMV CP, which delayed the onset of systemic symptoms compared to the WT after inoculation (Figure 1, Figure 3 and Figure 4). This directly indicated that V94A, V97, T104A, and E96A were capable of infecting *N. benthamiana* and watermelon, and that these mutations affected the infectivity of CGMMV.

**Figure 5:**
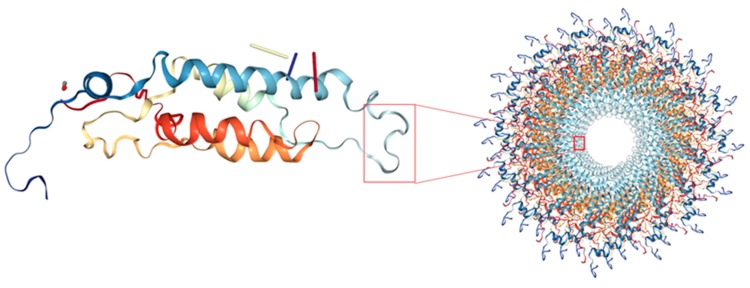
Three-dimensional model of the CGMMV coat protein. On the left is a CP subunit and on the right is the top view of the virion. The mutations are located within the red framed region.

To investigate the role of residue E96 in CGMMV pathogenicity, we established three double mutants in the background of the E96K mutation using the infectious cDNA clones of V94A, V97, and T104A. Subsequent experiments using an agroinfiltration assay and mechanical inoculation suggested that E96 was important for infecting *N. benthamiana*. Mutations of these CP residues are associated with virus infectivity and occurrence of host symptoms. These mutations in the gene encoding CP altered the sequence of both the RNA and amino acids of CP. However, we could not conclude whether these changes altered the pathogenesis of the virus. The initiation site for reconstitution of a watermelon strain of CGMMV was located approximately 320 nucleotides away from the 3′ terminus, and was within the CP cistron [22]. Furthermore, TMV virion assembly initiates at the initiation site and a two-way elongation was described previously [26,27]. Hence, all mutations introduced in the CP gene in this study were located at the assembly initiation site and the adjacent region, considering that the mutations may affect the efficiency of the assembly initiation site. Indeed, introduction of the E96K mutation in V94A, V97, and T104A mutants compensated the efficiency of the assembly initiation site at the RNA level. The 3D model of CGMMV (published by the Protein Database) shows that residues 94, 96, 97, and 104 covered the inner loop of the CGMMV CP subunit, which is located at the inner virion surface (Figure 5). In the absence of RNA, the structure of the inner loop is disordered, which allows access of the RNA to its binding site within the 20 S aggregate. However, RNA binding results in the structural ordering of residues within the inner loop, locking the nucleoprotein complex into a virion like helix, which allows progression of the assembly [28]. Therefore, the stability of the inner loop determines the efficiency of the virion assembly.

In a previous study, a series of TMV mutants with insertions and deletions in the CP allowed cell-to-cell movement but destroyed the assembly function and limited the systemic spread of the virus [29]. All inoculated leaves of the single and double mutants tested positive at 7 dpi by DAS-ELISA when V94A-E96K, V97-E96K, T104A-E96, and E96K-infected plants showed symptoms of infection (data not shown). This revealed that the mutations might inhibit the long-distance transport of the virus. Simon-Buela et al. [30] showed that CGMMV was transported in the phloem in the form of viral particles. However, whether the assembly of the CGMMV virion affects its systemic transport remains to be investigated. Notably, a previous study showed that a single amino acid mutation in the carnation ringspot virus CP allowed virion formation but prevented systemic infection [31]. MP and CP are required for cell-to-cell and long-distance transport of the virus, respectively. However, Bendahmane et al. [32] showed that a mutant CP interferes with MP accumulation and cell-to-cell movement of infection. Herein, the phenomenon of a delay in systemic symptom would be possible if the mutant CP has a negative effect on MP. However, we have no direct evidence of any relationship between accumulation of MP and CP in this study.

The occurrence of a spontaneous and non-anticipatory mutation in three different mutants is unlikely to be a random event. In our previous research, amino acid residue 284 of the replicase reversed Cys to Arg after inoculation. The reversions to wild type may play major roles in the stability of mutants [18]. According to previous studies in tobamoviruses, the mean evolutionary rates at the nucleotide and amino acid levels range between 1.0 × 10^−5^ and 1.3 × 10^−3^ substitutions per site per year, which are similar to those seen in a broad spectrum of animal and plant RNA viruses [33]. Amidst such high mutation rates and strong environmental selective forces, RNA viruses replicate as complex and dynamic mutant swarms, called viral quasispecies [34]. The occurrence of a mutation is random, but selective forces govern survival and reproduction. Therefore, the evolution of viral fitness has to be considered simultaneously with the selective forces. Currently, we are investigating the coincidence of viral fitness.

## Figures and Tables

**Table 1:** Primers used in site directed mutagenesis with Glu to Lys substitution at residue 96 of Cucumber green mottle mosaic virus (CGMMV) coat protein (CP).

Mutants	Primers	Sequences (5′-3′)
E96K	E96K-X	AAGGTTGTAGATCCTAGCAATCCCA
	E96K-S	TAGGATCTACAACCTTAATGACCCTA
V94A-E96K	V94A-E96K-X	AAGGTTGTAGATCCTAGCAATCCCA
	V94A-E96K-S	TAGGATCTACAACCTTAATAGCCCTA
V97-E96K	V97-E96K-X	AAGGTAGATCCTAGCAATCCCACGA
	V97-E96K-S	TGCTAGGATCTACCTTAATGACCCTA
T104A-E96K	T104A-E96K-X	CGTAATAGGGTCATTAAGGTTGTAGA
	T104A-E96K-S	TAATGACCCTATTACGCGTATCCGT

-X, downstream primer; -S, upstream primer.

**Table 2:** Systemic infection of *N. benthamiana* with CGMMV mutants and detection of upper non-inoculated leaves of *N. benthamiana* by double antibody sandwich enzyme-linked immunosorbent assay (DAS-ELISA).

Plant	Mutants	7 dpi			14 dpi			21 dpi		
		Symptoms	Y/N	ELISA	Symptoms	Y/N	ELISA	Symptoms	Y/N	ELISA
N.B	V94A-E96K	M	8/12	+	M, Ma	12/12	+	M	12/12	+
N.B	V94A	——	0/12	+	Ma	9/12	+	M	10/12	+
N.B	V97-E96K	M, Ma	7/12	+	M, Ma	12/12	+	M	12/12	+
N.B	V97	——	0/12	−	——	0/12	−	——	0/12	+
N.B	T104A-E96K	M	5/12	+	M, Ma	12/12	+	M	12/12	+
N.B	T104A	——	0/12	−	——	0/12	−	M, Ma	3/12	+
N.B	E96K	M, Ma	11/12	+	M, Ma	12/12	+	M	12/12	+
N.B	E96A	——	0/12	+	M, Ma	12/12	+	M	12/12	+
N.B	WT	M, Ma	6/12	+	M, Ma	12/12	+	M	12/12	+
N.B	CK	——	0/12	−	——	——	−	——	——	−

Y, number of systemically infected plants; N, number of plant inoculated; M, mosaic, Ma, malformation; N.B, N. benthamiana. ——, asymptomatic; +, positive in DAS-ELISA; −, negative in DAS-ELISA; WT: wild type (C284R); CK: blank control check.
